# When culture-based whole-genome sequencing can change management in severe ocular bacterial infections

**DOI:** 10.1128/mbio.00659-26

**Published:** 2026-04-13

**Authors:** Inès Fenniri, Camille André

**Affiliations:** 1 Department of Ophthalmology, Hospices Civils de Lyon 26900, Lyon, France; 2 Department of Ophthalmology, Boston Children’s Hospital, Harvard Medical School 1811 , Boston, Massachusetts, USA; 3 Department of Ophthalmology, Massachusetts Eye and Ear, Harvard Medical School 1811 , Boston, Massachusetts, USA; 4 Infectious Disease Institute, Massachusetts Eye and Ear, Harvard Medical School 1811 , Boston, Massachusetts, USA; The Ohio State University, Columbus, Ohio, USA

**Keywords:** antimicrobial resistance, eye infections, genomics, diagnostics

## Abstract

Nationwide multicenter surveillance demonstrates a substantial burden of antimicrobial resistance among ocular bacterial pathogens. In the 2009–2018 ARMOR collection, methicillin-resistant *Staphylococcus aureus* (MRSA) accounted for 34.9% of *S. aureus* ocular isolates, and methicillin resistance was strongly associated with fluoroquinolone resistance. Ocular infections can destroy tissue within hours; timely microbiologic diagnosis and treatment adjustment are essential; however, culture is constrained by small specimen volumes, low inoculum, and prior antimicrobial exposure. To tackle multidrug-resistant (MDR) infections, we propose that phenotypic testing could be complemented with targeted whole-genome sequencing in cases of severe or persistent eye infections, to provide lineage characterization, resistance and virulence determinants, and critical features of pathogenic MDR bacteria that are clinically relevant to predict patient outcomes. A genome-informed workflow, sequencing from pure culture, and integration with phenotypic data could refine empiric therapy, enable earlier de-escalation, and strengthen surveillance of high-risk lineages to reduce vision-threatening treatment failures and save patients’ vision.

## PERSPECTIVE

Ocular infections are time-sensitive and anatomically unforgiving. They highlight the growing concern of multidrug-resistant (MDR) bacteria, particularly in ophthalmology, where empiric regimens can be mismatched due to local resistance patterns (1, 2).

Infectious keratitis is widely recognized as a leading cause of corneal blindness worldwide and remains a major source of preventable vision loss (3–5). Outcomes are often determined by a time-sensitive therapeutic window, in which early effective antimicrobial therapy distinguishes recovery from irreversible corneal opacity (6, 7). Endophthalmitis, although less common, is among the most vision-threatening ophthalmic emergencies; delayed or ineffective therapy can rapidly translate into permanent visual disability (8, 9). Across these sight-threatening infections, gram-positive organisms—particularly *Staphylococcus aureus* and *Streptococcus pneumoniae*—are frequent causes, and outcomes depend on a narrow window for effective therapy (8, 10). Among gram-negative organisms, *Pseudomonas aeruginosa* also contributes substantially and is a recurring isolate identified in keratitis cases (3, 4, 10, 11).

## AMR BURDEN IN OPHTHALMOLOGY

Nationwide multicenter surveillance programs document substantial antibiotic resistance among ocular pathogens, particularly methicillin-resistant *S. aureus* (MRSA) (12, 13). In the ARMOR study conducted in the U.S. from 2009 to 2018, MRSA accounted for 34.9% of ocular *S. aureus* isolates, and fluoroquinolone resistance was markedly higher in MRSA than in methicillin-susceptible *S. aureus* (e.g., ciprofloxacin 72.7% vs. 10.4%; levofloxacin 69.4% vs. 9.9%) (13). Fluoroquinolones remain widely used first-line agents in ophthalmology, and their widespread use has likely contributed to selection and maintenance of high-level resistance, particularly in MRSA (4, 7, 13). Earlier national surveillance (2000–2005) similarly documented increasing methicillin resistance among serious ocular *S. aureus* infections (14). This has direct clinical relevance: in a tissue-limited organ, resistance-driven treatment failures can rapidly result in irreversible damage, including blindness (8, 9, 15, 16). Consistent with this risk, the WHO Global Action Plan on antimicrobial resistance (AMR) emphasizes surveillance and research as central pillars to counter resistance (17, 18). In ophthalmology, AMR leading to therapeutic failure may translate directly into permanent visual loss (6, 8, 9).

Phenotypic surveillance remains fundamental in ophthalmology: antibiotic minimum inhibitory concentrations (MICs) define *in vitro* levels of susceptibility or resistance of specific bacterial strains to applied antibiotics and are indispensable for establishing prophylaxis and stewardship protocols (2, 13). However, phenotype does not distinguish whether resistance is broadly distributed or concentrated within a small number of high-risk clones (19, 20). For example, whole-genome sequencing (WGS) has revealed that specific *Klebsiella pneumoniae* lineages, particularly hypervirulent K1/ST23 strains, carry virulence determinants, such as *rmpA* and *magA*, and harbor extended-spectrum β-lactamase genes, which are associated with invasive disease and metastatic complications, including endogenous endophthalmitis, a condition frequently resulting in poor visual outcomes (21). This distinction is critical in time-sensitive ocular infections, where empiric decisions are made before MIC results are available, and where clone-specific resistance profiles and virulence may plausibly influence both treatments and prognosis (19, 22). In this context, this Perspective discusses the potential implementation of culture-based WGS as an adjunct to routine microbiology for severe ocular infections and suspected outbreak clusters (19, 20).

## DIAGNOSTIC CONSTRAINTS IN OCULAR INFECTION: WHY “CULTURE FIRST” IS NECESSARY BUT INSUFFICIENT

Ocular infection diagnostics face three recurring constraints. First, specimens are tiny and often irreplaceable (23). Second, bacterial inocula are frequently low, particularly after partial antimicrobial treatment (23). Third, pre-sampling antimicrobial exposure is common, because clinicians must initiate therapy immediately when vision is at risk (6, 7, 23). Even when cultures are positive, turnaround time for organism identification and susceptibility may extend beyond the earliest decisive clinical window (23). Practical guidance emphasizes that sampling technique, transport, and laboratory workflows can improve sensitivity, but they cannot overcome these underlying biological constraints (23). As a result, empiric broad-spectrum therapy is often initiated before microbiologic results become available, and sampling practices remain variable across settings (7, 23). While rational at the bedside, this approach increases selection pressure and can entrench local resistance patterns over time (1, 2, 17). In the absence of culture and susceptibility results, resistance-surveillance data can inform empiric choices; nationwide initiatives such as ARMOR track resistance to drugs commonly used in ophthalmology (12, 13). However, most surveillance remains limited to phenotypic susceptibility and does not include routine molecular characterization of ocular isolates, underscoring the need for complementary genomic approaches (2, 20).

## WHAT CULTURE-BASED WGS ADDS BEYOND CULTURE AND OTHER MOLECULAR DIAGNOSTICS

As culture may be time-consuming and poorly sensitive for the diagnosis of ocular infections, PCR-based assays have been used to improve the sensitivity of pathogen detection (24, 25) (Table 1). The advantages of PCR include its speed and sensitivity, including the detection of slow-growing bacteria that are traditionally difficult to cultivate or identify with traditional microbiological methods (26, 27). On the other hand, the disadvantages include the high rate of false-positive errors from commensal contaminants or dead bacteria, lower specificity compared with culture, and the need to narrow the list of causative agents to use specific primers.

**TABLE 1:** Comparative roles of culture, phenotypic AST, molecular assays, metagenomic sequencing, and culture-based WGS in ocular bacterial infections^*a*^

Approach	Workflow position/typical TAT^*b*^	Main strengths	Main limitations
Culture + phenotypic AST (4, 7)	First-line after corneal or intraocular sampling; growth-dependent (typically days)	Clinical standard; provides isolate for confirmatory testing and downstream analyses	Poor sensitivity after prior antibiotics or low biomass; no resistance mechanism, no lineage characterization, or genomic relatedness
Targeted PCR or 16S PCR (28, 29)	Direct from sample; several hours to same day	Rapid answer to a focused question; useful when prior therapy may reduce culture yield or when culture is negative	Require an a priori hypothesis; targeted PCR narrows scope; no isolate for AST, false-positive errors from commensal or dead bacteria
Metagenomic NGS (30–32)	Direct from sample; specialized workflow, often 1-3 days	Unbiased detection of multiple pathogens, including fastidious organisms, or culture-negative case	High cost; host-background signal; complex interpretation; limited direct susceptibility information
Culture-based WGS (22, 33–38)	Performed only after isolate recovery in specialized settings; adds sequencing, and bioinformatics analysis (48-72 h)	Species-specific lineage characterization, identification of resistance, virulence genes, and supports cluster investigation	Costly, requires viable isolate, and dedicated sequencing, bioinformatics, and infrastructure

^*a*^ AST, antimicrobial susceptibility testing; NGS, next-generation sequencing; TAT, turnaround time; WGS, whole-genome sequencing.

^*b*^ Turnaround times depend on specimen quality, organism growth, batching strategy, sequencing platform, and local laboratory workflow (28, 29, 33, 38).

Whole-genome sequencing started to be incorporated in clinical microbiology and hospital epidemiology to provide high-resolution strain typing, transmission reconstruction, and clone-level risk stratification, particularly for MRSA and other high-burden pathogens (19, 20). In practice, WGS is more commonly implemented in reference laboratories and specialized centers such as the Centers for Disease Control and Prevention (CDC), where it is used for purposes such as outbreak investigation, genomic surveillance, and characterization of antimicrobial resistance mechanisms, especially for systemic infections (19, 20, 39).

Ophthalmology remains comparatively phenotype-forward: surveillance and management of eye infections rarely include systematic lineage characterization of ocular isolates (2, 13). Although culture and phenotypic antimicrobial susceptibility testing (AST) are indispensable for isolate recovery, species identification, and susceptibility interpretation (7, 23, 40), they do not answer every clinically relevant question once an isolate has been recovered (20, 33, 39). In selected severe ocular bacterial infections, the remaining question may be whether an isolate carries a resistance mechanism, belongs to a species-specific lineage enriched for multidrug resistance or poor visual outcomes, or is genomically related to other isolates in a way that supports a common source or healthcare-associated transmission (19, 22, 34, 41, 42). These are the specific gaps in which culture-based WGS may add value.

Early ophthalmic WGS studies have already reported clinically relevant patterns in staphylococcal disease, with recurrent lineages such as CC8 and CC5 carrying distinct multidrug-resistant profiles and exhibiting distinct ocular tissue tropisms (34, 41). CC5 MDR MRSA strains are more frequently associated with keratitis, while CC8 strains are more commonly associated with periocular infections (10, 34, 41). In severe exogenous MRSA endophthalmitis (22) and keratitis (43), multidrug-resistant lineages have been associated with poor outcomes, suggesting that genomic context may be clinically informative rather than merely descriptive. For pneumococcal conjunctivitis, WGS has defined a distinct phylogenetic cluster of unencapsulated isolates with new virulence factors that grouped within the epidemic conjunctivitis cluster (44, 45). By contrast, pneumococcal keratitis is caused by a highly diverse population composed largely of non-vaccine serotypes (46). Gram-negative eye infections are also amenable to this approach. WGS analyses of ocular *P. aeruginosa* isolates demonstrate substantial genomic diversity, supporting the value of local lineage characterization to anticipate resistance patterns and to guide empiric escalation with early de-escalation when phenotypic susceptibility permits (11).

A recent ocular report described keratitis cases caused by MDR *P. aeruginosa* carrying *blaGES* and *blaVIM* carbapenemase-resistance genes (47) that were resistant to first-line topical therapy. Moreover, WGS recently identified a cluster of postoperative *P. aeruginosa* endophthalmitis at an outpatient cataract surgery center (48).

**Fig 1:**
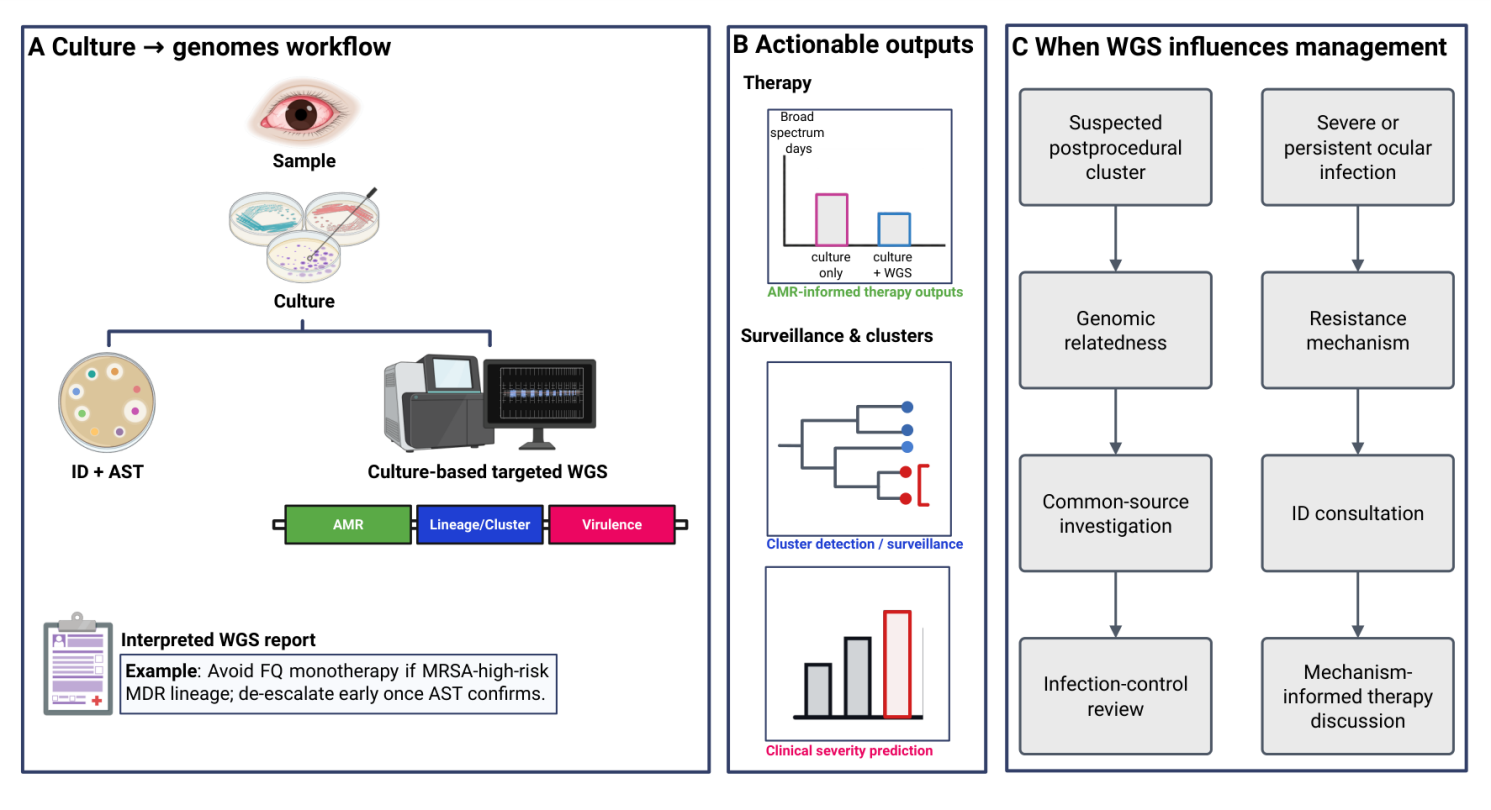
From culture to genomes: a genome-informed pathway for ocular infections in the antimicrobial resistance era. (**A**) Culture → genomes workflow. Clinical sampling is followed by culture, enabling two complementary laboratory paths. Routine microbiology provides organism identification and phenotypic antimicrobial susceptibility testing (ID + AST). In parallel, WGS from pure culture adds three interpretable layers: AMR determinants, lineage/cluster characterization, and virulence features. A brief interpreted WGS report will translate genomic findings into a statement to guide therapy. (**B**) Actionable outputs. Therapy: genomic AMR context is used to support earlier narrowing and potentially reduce broad-spectrum exposure compared with culture alone. Surveillance and clusters: lineage-phylogenetic relatedness supports tracking predominant clones and flagging clusters for infection-control review. Integrating lineage and virulence factors can further establish a score to predict clinical severity stratification. (**C**) When WGS influences management. Abbreviations: AMR, antimicrobial resistance; AST, antimicrobial susceptibility testing; WGS, whole-genome sequencing; ID, identification; MRSA, methicillin-resistant *Staphylococcus aureus*; MDR, multidrug-resistant; FQ, fluoroquinolone. Created in BioRender (I. Fenniri, 2026, https://BioRender.com/7fjmn0d, licensed under CC BY 4.0).

We therefore suggest three situations in which culture-based WGS is most plausibly actionable: severe bacterial keratitis threatening perforation or central scarring, culture-positive endophthalmitis, and suspected postoperative or intravitreal injection-associated clusters (6, 8, 19, 33, Fig. 1).

## WHAT AN OPHTHALMOLOGY-FACING WGS REPORT MAY CONTAIN

For WGS to be clinically relevant in ophthalmology, the output should be a brief interpreted report rather than a raw gene list (35). It must be a short, clinician-oriented report that privileges interpretation, states limitations explicitly, and aligns with routine microbiology workflows (20, 39). First, the report should assign lineage (MLST-derived sequence type and clonal complex) and translate that designation into one sentence of clinical meaning—whether the lineage is historically associated with multidrug resistance, frequently implicated in severe ocular infection, or relevant to local epidemiology (19, 34). Second, it should provide a comprehensible resistome report that includes acquired resistance genes and chromosomal mutations stratified by antibiotic classes. It should also distinguish “determinant detected” from “no determinant detected” and avoid any implication that genotype replaces phenotypic susceptibility testing (20, 35, 36, 39). Third, when relevant, it should report genomic relatedness to other isolates in the context of a suspected cluster (37). Finally, the report should include a short interpretive note stating what the genomic data may add, what they do not establish, and whether infection-control review should be considered (35, 37). Because such reports require interpretation beyond standard microbiology outputs, implementation also depends on clinician genomic training and close collaboration among ophthalmology, infectious diseases, and clinical microbiology teams (19, 37).

## IMPLEMENTATION AND LIMITATIONS

The goal is not to sequence everything, but to focus on situations in which genomics can change management, such as severe keratitis at risk of perforation or central scarring, culture-positive endophthalmitis, refractory infections, and suspected procedural or ward-linked clusters (6–8, 19).

A small sampling of routine culture-positive cases can then sustain local lineage surveillance without being excessively costly for the hospital (19, 20).

As discussed above, WGS becomes clinically relevant only when returned as a brief interpreted report that complements phenotype (lineage assignment, resistance, and virulence determinants) (20, 34, 39). ISO-certified clinical genomics workflows have already shown that AMR determinants can be binned by antibiotic class and translated into customized per-isolate reports, supporting the feasibility of an ophthalmology-facing resistome summary rather than a raw gene list (36).

With appropriate interpretation, WGS can inform empiric treatment adjustment and support earlier narrowing of antimicrobial therapy (22).

A fair critique is necessary as culture-based WGS depends on culture positivity, adds cost, and does not rescue culture-negative cases (20, 23). Culture-based WGS is not faster than initial culture and, in many settings, may not be faster than routine AST (20, 33). Targeted ocular PCR can provide a rapid answer in selected settings (28). WGS implementation also requires standardized pipelines, bioinformatics expertise, quality control, and clinician education (33, 35–37). These requirements remain substantial barriers to routine deployment outside selected use cases (20, 33, 37). Accordingly, early implementation should be tied to a prespecified metric set that captures turnaround, antimicrobial stewardship, vision-relevant outcomes, and cluster detection. A pragmatic core set may include speed (time to appropriate therapy and time to de-escalation) (6, 7, 49), antibiotic pressure (days of broad-spectrum therapy) (1, 17), vision-relevant outcomes (best-corrected visual acuity at a fixed time point, therapeutic keratoplasty for keratitis, and evisceration/enucleation for endophthalmitis) (7, 8, 15), and epidemiology (time to cluster detection) (19). Implementation can start with a single center using predefined indications and a standardized report template and then expand to multicenter harmonization once feasibility is established (19, 20).

## CONCLUSION

To tackle the threat of MDR bacteria causing eye infections, a shift toward molecular characterization of these pathogens is essential. The importance of including WGS in ophthalmology is that it provides new and critical insights that will allow eye care professionals to manage infections in a way that reduces the likelihood of sight-threatening infections by identifying biomarkers and critical features of pathogenic MDR bacteria lineages that can be targeted therapeutically to save patients’ vision. Combined with culture, it can provide critical complementary information, including predominant circulating lineages and associated acquired AMR, with simple alerts when clusters emerge. That will allow empiric protocols to evolve from habit into evidence, updated to the lineages currently circulating in a given center or region.

## References

[1] Cabrera-Aguas M, Chidi-Egboka N, Kandel H, Watson SL. 2024. Antimicrobial resistance in ocular infection: a review. Clin Exp Ophthalmol 52:258–275. doi:10.1111/ceo.14377 38494451

[2] Bispo PJM, Sahm DF, Asbell PA. 2022. A systematic review of multi-decade antibiotic resistance data for ocular bacterial pathogens in the United States. Ophthalmol Ther 11:503–520. doi:10.1007/s40123-021-00449-9 35113406 PMC8927494

[3] Ting DSJ, Ho CS, Deshmukh R, Said DG, Dua HS. 2021. Infectious keratitis: an update on epidemiology, causative microorganisms, risk factors, and antimicrobial resistance. Eye (London) 35:1084–1101. doi:10.1038/s41433-020-01339-3 PMC8102486 33414529

[4] Cabrera‐Aguas M, Khoo P, Watson SL. 2022. Infectious keratitis: a review. Clinical Exper Ophthalmology 50:543–562. doi:10.1111/ceo.14113 PMC9542356 35610943

[5] Whitcher JP, Srinivasan M, Upadhyay MP. 2001. Corneal blindness: a global perspective. Bull World Health Organ 79:214–221. doi:10.1590/S0042-96862001000300009 11285665 PMC2566379

[6] Ung L, Chodosh J. 2023. Urgent unmet needs in the care of bacterial keratitis: an evidence-based synthesis. Ocul Surf 28:378–400. doi:10.1016/j.jtos.2021.08.013 34461290 PMC10721114

[7] Rhee MK, Ahmad S, Amescua G, Cheung AY, Choi DS, Jhanji V, Lin A, Mian SI, Viriya ET, Mah FS, Varu DM. 2024. Bacterial keratitis preferred practice pattern. Ophthalmology 131:P87–P133. doi:10.1016/j.ophtha.2023.12.03537598860

[8] Durand ML. 2017. Bacterial and fungal endophthalmitis. Clin Microbiol Rev 30:597–613. doi:10.1128/CMR.00113-16 28356323 PMC5475221

[9] Novosad BD, Callegan MC. 2010. Severe bacterial endophthalmitis: towards improving clinical outcomes. Expert Rev Ophthalmol 5:689–698. doi:10.1586/eop.10.52 21572565 PMC3092298

[10] André C, Lebreton F, Van Tyne D, Cadorette J, Boody R, Gilmore MS, Bispo PJM. 2023. Microbiology of eye infections at the Massachusetts eye and ear: an 8-year retrospective review combined with genomic epidemiology. Am J Ophthalmol 255:43–56. doi:10.1016/j.ajo.2023.06.016 37343741 PMC10592486

[11] Miranda SW, André C, Bispo PJM, Gilmore MS. 2025. Whole genome analysis of ocular Pseudomonas aeruginosa isolates reveals genetic diversity. Invest Ophthalmol Vis Sci 66:58. doi:10.1167/iovs.66.6.58 PMC12180601 40530921

[12] Haas W, Pillar CM, Torres M, Morris TW, Sahm DF. 2011. Monitoring antibiotic resistance in ocular microorganisms: results from the antibiotic resistance monitoring in ocular microrganisms (ARMOR) 2009 surveillance study. Am J Ophthalmol 152:567–574. doi:10.1016/j.ajo.2011.03.010 21652021

[13] Asbell PA, Sanfilippo CM, Sahm DF, DeCory HH. 2020. Trends in antibiotic resistance among ocular microorganisms in the United States from 2009 to 2018. JAMA Ophthalmol 138:439–450. doi:10.1001/jamaophthalmol.2020.0155 32271355 PMC7146550

[14] Asbell PA, Sahm DF, Shaw M, Draghi DC, Brown NP. 2008. Increasing prevalence of methicillin resistance in serious ocular infections caused by Staphylococcus aureus in the United States: 2000 to 2005. J Cataract Refract Surg 34:814–818. doi:10.1016/j.jcrs.2008.01.016 18471638

[15] Lu X, Ng D-C, Zheng K, Peng K, Jin C, Xia H, Chen W, Chen H. 2016. Risk factors for endophthalmitis requiring evisceration or enucleation. Sci Rep 6:28100. doi:10.1038/srep28100 27302573 PMC4908388

[16] Song X, Xie L, Tan X, Wang Z, Yang Y, Yuan Y, Deng Y, Fu S, Xu J, Sun X, Sheng X, Wang Q. 2014. A multi-center, cross-sectional study on the burden of infectious keratitis in China. PLoS One 9:e113843. doi:10.1371/journal.pone.0113843 25438169 PMC4250054

[17] World Health Organization. 2015. Global action plan on antimicrobial resistance. Geneva World Health Organization

[18] Murray CJL, Ikuta KS, Sharara F, Swetschinski L, Robles Aguilar G, Gray A, Han C, Bisignano C, Rao P, Wool E, et al.. 2022. Global burden of bacterial antimicrobial resistance in 2019: a systematic analysis. Lancet 399:629–655. doi:10.1016/S0140-6736(21)02724-0 35065702 PMC8841637

[19] Humphreys H, Coleman DC. 2019. Contribution of whole-genome sequencing to understanding of the epidemiology and control of meticillin-resistant Staphylococcus aureus. J Hosp Infect 102:189–199. doi:10.1016/j.jhin.2019.01.025 30721732

[20] Tagini F, Greub G. 2017. Bacterial genome sequencing in clinical microbiology: a pathogen-oriented review. Eur J Clin Microbiol Infect Dis 36:2007–2020. doi:10.1007/s10096-017-3024-6 28639162 PMC5653721

[21] Xu M, Li A, Kong H, Zhang W, Chen H, Fu Y, Fu Y. 2018. Endogenous endophthalmitis caused by a multidrug-resistant hypervirulent Klebsiella pneumoniae strain belonging to a novel single locus variant of ST23: first case report in China. BMC Infect Dis 18:669. doi:10.1186/s12879-018-3543-5 30558549 PMC6296127

[22] Ali FZA, Andre C, Sobrin L, Sun J, Boody R, Cadorette J, Bispo PJM. 2025. Exogenous methicillin-resistant Staphylococcus aureus endophthalmitis is caused by multidrug-resistant lineages that are associated with poor outcomes. Ocul Immunol Inflamm 33:446–456. doi:10.1080/09273948.2024.2417797 39446740

[23] Leal SM, Rodino KG, Fowler WC, Gilligan PH. 2021. Practical guidance for clinical microbiology laboratories: diagnosis of ocular infections. Clin Microbiol Rev 34:e00070-19. doi:10.1128/CMR.00070-19 34076493 PMC8262805

[24] Bispo PJM, Davoudi S, Sahm ML, Ren A, Miller J, Romano J, Sobrin L, Gilmore MS. 2018. Rapid detection and identification of uveitis pathogens by qualitative multiplex real-time PCR. Invest Ophthalmol Vis Sci 59:582–589. doi:10.1167/iovs.17-22597 29372257 PMC5788046

[25] Kosacki J, Boisset S, Maurin M, Cornut P-L, Thuret G, Hubanova R, Vandenesch F, Carricajo A, Aptel F, Chiquet C, Friends Group. 2020. Specific PCR and quantitative real-time PCR in ocular samples from acute and delayed-onset postoperative endophthalmitis. Am J Ophthalmol 212:34–42. doi:10.1016/j.ajo.2019.11.026 31770517

[26] Taravati P, Lam D, Van Gelder RN. 2013. Role of molecular diagnostics in ocular microbiology. Curr Ophthalmol Rep 1:181–189. doi:10.1007/s40135-013-0025-1 PMC3885281 24416712

[27] Liu HY, Hopping GC, Vaidyanathan U, Ronquillo YC, Hoopes PC, Moshirfar M. 2019. Polymerase chain reaction and its application in the diagnosis of infectious keratitis. Med Hypothesis Discov Innov Ophthalmol 8:152–155. 31598517 PMC6778471

[28] Sugita S, Takase H, Nakano S. 2023. Role of recent PCR tests for infectious ocular diseases: from laboratory-based studies to the clinic. Int J Mol Sci 24:8146. doi:10.3390/ijms24098146 37175854 PMC10179472

[29] Somerville TF, Corless CE, Sueke H, Neal T, Kaye SB. 2020. 16S ribosomal RNA PCR versus conventional diagnostic culture in the investigation of suspected bacterial keratitis. Transl Vis Sci Technol 9:2. doi:10.1167/tvst.9.13.2 PMC7718820 33344046

[30] Lalitha P, Prajna NV, Sikha M, Gunasekaran R, Hinterwirth A, Worden L, Chen C, Zhong L, Liu Z, Lietman TM, Seitzman GD, Doan T. 2021. Evaluation of metagenomic deep sequencing as a diagnostic test for infectious keratitis. Ophthalmology 128:473–475. doi:10.1016/j.ophtha.2020.07.030 32682834 PMC7856230

[31] Pan X-Y, Wang M, Xu Y-D, Wang L-N. 2024. Application of metagenomic next-generation sequencing in the diagnosis of infectious keratitis. J Ophthalmol 2024:9911979. doi:10.1155/2024/9911979 38716089 PMC11074721

[32] Seitzman GD, Hinterwirth A, Zhong L, Cummings ME, Chen C, Driver TH, Lee MD, Doan T. 2019. Metagenomic deep sequencing for the diagnosis of corneal and external disease infections. Ophthalmology 126:1724–1726. doi:10.1016/j.ophtha.2019.06.013 31421897

[33] Rossen JWA, Friedrich AW, Moran-Gilad J. 2018. Practical issues in implementing whole-genome-sequencing in routine diagnostic microbiology. Clin Microbiol Infect 24:355–360. doi:10.1016/j.cmi.2017.11.001 29117578

[34] André C, Van Camp AG, Ung L, Gilmore MS, Bispo PJM. 2024. Characterization of the resistome and predominant genetic lineages of gram-positive bacteria causing keratitis. Antimicrob Agents Chemother 68:e01247-23. doi:10.1128/aac.01247-23 38289077 PMC10916405

[35] Mutschler E, Roloff T, Neves A, Vangstein Aamot H, Rodriguez-Sanchez B, Ramirez M, Rossen J, Couto N, Novais Â, Howden BP, Brisse S, Reuter S, Nolte O, Egli A, Seth-Smith HMB, ESCMID Study Group for Epidemiological Markers (ESGEM), and ESCMID Study Group for Genomic and Molecular Diagnostics (ESGMD). 2024. Towards unified reporting of genome sequencing results in clinical microbiology. PeerJ 12:e17673. doi:10.7717/peerj.17673 39131622 PMC11317035

[36] Sherry NL, Horan KA, Ballard SA, Gonҫalves da Silva A, Gorrie CL, Schultz MB, Stevens K, Valcanis M, Sait ML, Stinear TP, Howden BP, Seemann T. 2023. An ISO-certified genomics workflow for identification and surveillance of antimicrobial resistance. Nat Commun 14:60. doi:10.1038/s41467-022-35713-4 36599823 PMC9813266

[37] Sundermann AJ, Rosa R, Harris PNA, Snitkin E, Javaid W, Moore NM, Hayden MK, Allen K, Rodino K, Peacock SJ, Abbo LM, Harrison LH. 2025. Pathogen genomics in healthcare: overcoming barriers to proactive surveillance. Antimicrob Agents Chemother 69:e01479-24. doi:10.1128/aac.01479-24 39636107 PMC11784250

[38] Kwong JC, McCallum N, Sintchenko V, Howden BP. 2015. Whole genome sequencing in clinical and public health microbiology. Pathology (Philadelphia) 47:199–210. doi:10.1097/PAT.0000000000000235 PMC4389090 25730631

[39] Price J, Claire Gordon N, Crook D, Llewelyn M, Paul J. 2013. The usefulness of whole genome sequencing in the management of Staphylococcus aureus infections. Clin Microbiol Infect 19:784–789. doi:10.1111/1469-0691.12109 23331482

[40] Ting DSJ, Gopal BP, Deshmukh R, Seitzman GD, Said DG, Dua HS. 2022. Diagnostic armamentarium of infectious keratitis: a comprehensive review. Ocul Surf 23:27–39. doi:10.1016/j.jtos.2021.11.003 34781020 PMC8810150

[41] Bispo PJM, Ung L, Chodosh J, Gilmore MS. 2020. Hospital-associated multidrug-resistant MRSA lineages are trophic to the ocular surface and cause severe microbial keratitis. Front Public Health 8:204. doi:10.3389/fpubh.2020.00204 32582610 PMC7283494

[42] Tuft S, Somerville TF, Li J-P, Neal T, De S, Horsburgh MJ, Fothergill JL, Foulkes D, Kaye S. 2022. Bacterial keratitis: identifying the areas of clinical uncertainty. Prog Retin Eye Res 89:101031. doi:10.1016/j.preteyeres.2021.101031 34915112

[43] Cummings OW, Andre C, Ling J, Yeung S, Wong T, Iovieno A, Bispo PJM. 2023. Methicillin-resistant Staphylococcus aureus keratitis is mainly caused by multidrug-resistant lineages that are associated with poor outcomes. Invest Ophthalmol Vis Sci 64:2329.

[44] Valentino MD, McGuire AM, Rosch JW, Bispo PJM, Burnham C, Sanfilippo CM, Carter RA, Zegans ME, Beall B, Earl AM, Tuomanen EI, Morris TW, Haas W, Gilmore MS. 2014. Unencapsulated Streptococcus pneumoniae from conjunctivitis encode variant traits and belong to a distinct phylogenetic cluster. Nat Commun 5:5411. doi:10.1038/ncomms6411 25388376 PMC4231546

[45] Ung L, Bispo PJM, Bryan NC, Andre C, Chodosh J, Gilmore MS. 2019. The best of all worlds: Streptococcus pneumoniae conjunctivitis through the lens of community ecology and microbial biogeography. Microorganisms 8:46. doi:10.3390/microorganisms8010046 31881682 PMC7022640

[46] Andre C, Rouhana J, Scarpa de Mello S, Rosa da Cunha G, Van Camp AG, Gilmore MS, Bispo PJM. 2022. Population structure of ocular Streptococcus pneumoniae is highly diverse and formed by lineages that escape current vaccines. Microb Genom 8:000763. doi:10.1099/mgen.0.000763 35254235 PMC9176286

[47] Tribin FE, Lieux C, Maestre-Mesa J, Durkee H, Krishna K, Chou B, Neag E, Tóthová JD, Martinez JD, Flynn HW, Parel JM, Miller D, Amescua G. 2024. Clinical features and treatment outcomes of carbapenem-resistant Pseudomonas aeruginosa keratitis. JAMA Ophthalmol 142:407–415. doi:10.1001/jamaophthalmol.2024.0259 38512246 PMC10958388

[48] Singanamala S, Incekara K, Nucci D, Roberts SC. 2024. A cluster of postoperative Pseudomonas aeruginosa endophthalmitis infection at an outpatient cataract surgery center. Infect Control Hosp Epidemiol:1–2. doi:10.1017/ice.2024.74 38695341

[49] Ung L, Chodosh J. 2021. Urgent unmet needs in the care of bacterial keratitis: an evidence-based synthesis. Ocul Surf 28:378–400. doi:10.1016/j.jtos.2021.08.013 34461290 PMC10721114

